# Increased prevalence of depressive symptoms and stress in patients with chronic bothersome tinnitus during the COVID-19 pandemic

**DOI:** 10.1007/s00405-026-10553-w

**Published:** 2026-09-02

**Authors:** Sang-Yoon Han, Young Ho Kim

**Affiliations:** 1 Department of Otolaryngology-Head and Neck Surgery, College of Medicine, Hanyang University, Seoul, Republic of Korea; 2 Department of Otorhinolaryngology, Seoul National University Boramae Medical Center, Seoul National University College of Medicine, 5 Gil 20, Boramae-Road, Dongjak-Gu, Seoul, 156-707 Republic of Korea; 3 Department of Otorhinolaryngology-Head and Neck Surgery, Boramae Medical Center, SMG-SNU, Seoul, Republic of Korea

**Keywords:** COVID-19, Chronic tinnitus, Depressive symptoms, Stress, Psychological distress

## Abstract

**Objectives:**

The COVID-19 pandemic has been linked to various psychological and physical health consequences; however, its impact on tinnitus and associated psychological distress remains unexplored. This study evaluated the association between severity of chronic tinnitus and psychological outcomes, comparing these outcomes between the COVID-19 restriction period (2020) and the living-with-COVID-19 period (2022).

**Methods:**

Data were drawn from the 8th (2020) and 9th (2022) Korea National Health and Nutrition Examination Survey. A total of 5,965 participants were classified into bothersome tinnitus (visual analogue scale [VAS] > 4), non-bothersome tinnitus (VAS ≤ 4), and non-tinnitus groups. Depressive symptoms were assessed using the Patient Health Questionnaire-9 (PHQ-9), with a cut-off score of 5. Complex sample analyses were performed using sampling weights.

**Results:**

During the COVID-19 restriction period, the bothersome tinnitus group demonstrated significantly higher PHQ-9 scores and greater prevalence of depressive symptoms and high stress than the non-tinnitus group (*p* < 0.001 for all) and the non-bothersome tinnitus group (*p* < 0.05 for all). No significant differences were observed among groups during the living-with-COVID-19 period. Significant differences between the two periods were identified in the bothersome tinnitus group (PHQ-9, *p* = 0.048; depressive symptoms, *p* = 0.009; high stress, *p* = 0.039).

**Conclusion:**

Bothersome chronic tinnitus was independently associated with higher prevalence of depressive symptoms and high stress during the COVID-19 restriction period. These findings highlight the importance of tinnitus severity assessment for identifying psychological vulnerability and the need for integrated tinnitus and psychological management, including non-face-to-face approaches, when in-person access is restricted.

## Introduction

The COVID-19 pandemic profoundly disrupted societies worldwide [1, 2]. In South Korea, strict social distancing policies were implemented to control viral transmission. Although these measures were effective in reducing the number of early COVID-19 infections, both the social restrictions imposed during the pandemic and the burden of the disease itself led to a wide range of physical and psychological problems [3, 4]. Previous studies have suggested that the prevalence of depressive symptoms and anxiety increased during the pandemic period, while appropriate treatment and management of these conditions were often insufficient [3]. Tinnitus is also considered one of the representative conditions potentially affected by these circumstances.

Tinnitus can generally be classified into acute and chronic forms [5–7]. Tinnitus may arise from various etiologies, is frequently accompanied by hearing loss, and is closely associated with multiple psychological conditions [5–7]. When tinnitus becomes chronic, alterations in brain networks become established, and networks associated with various psychological conditions also undergo changes [8–14]. In particular, depressive symptom is one of the most common comorbidities in patients with chronic tinnitus and is known to exacerbate tinnitus severity [6, 7, 15]. Some other studies have reported a high prevalence of depression among individuals with chronic tinnitus [6, 7, 15]. Furthermore, neuroanatomical structures associated with depression have been identified within neural networks related to chronic tinnitus [8–14]. Therefore, patients with chronic tinnitus may respond differently to the psychological burden and “COVID-19 blues” associated with the pandemic compared with the general population.

Previous studies have demonstrated that chronic tinnitus is associated with stressful life events [16–19]. However, little research has examined whether individuals with chronic tinnitus exhibit greater vulnerability to stress and depressive symptoms in environments characterized by severe psychosocial stressors and social restrictions, such as those imposed by the COVID-19 pandemic.

Accordingly, this study aimed to investigate differences in stress and depressive symptoms according to chronic tinnitus severity using a nationally representative database during period of strict social distancing and during the subsequent easing of restrictions in the “living with COVID-19” phase. In addition, we sought to evaluate whether the presence and severity of chronic tinnitus were associated with increased depressive symptoms and stress related to social distancing measures.

## Materials and methods

### Data base, study subjects, and grouping

This study drew upon data from the 2020 and 2022 surveys of the eighth and ninth cycles, respectively, of the Korea National Health and Nutrition Examination Survey, (KNHANES) a nationally representative government-administered surveillance program. Established in 1998 and conducted on an annual basis since 2007 under the auspices of the Korea Disease Control and Prevention Agency (KDCA), KNHANES was designed to systematically monitor and evaluate the health status and nutritional intake of the South Korean general population. For the eighth and ninth survey cycles, a stratified sampling framework was employed, with primary stratification defined by province or metropolitan city, administrative unit type (dong/eup/myeon), and housing category. Additional implicit stratification was introduced through the systematic arrangement of sampling units according to demographic and socioeconomic characteristics, including sex, age, residential area composition, and the educational attainment of the head of household.

**Fig. 1:**
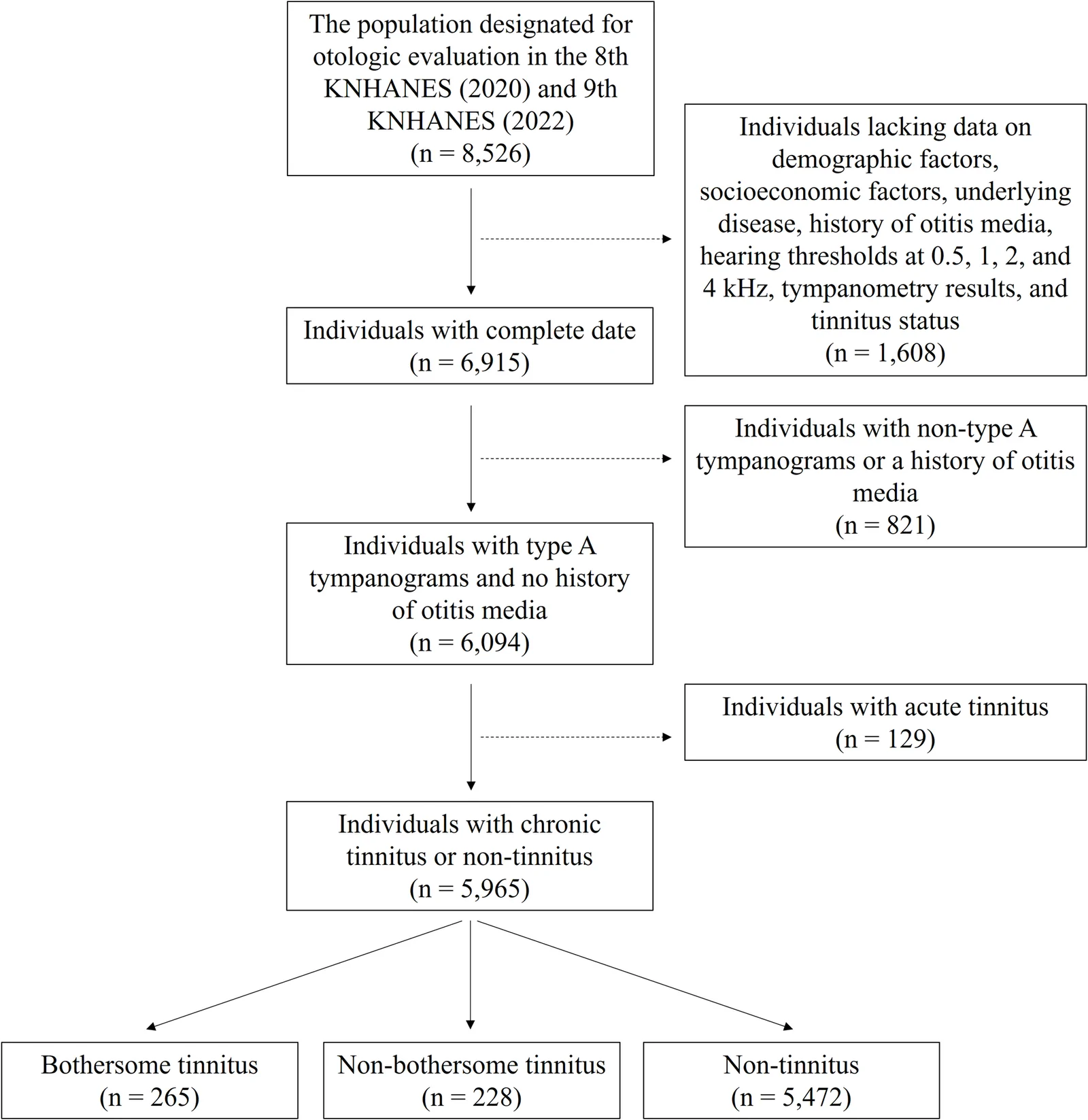
Flowchart of subject inclusion and exclusion criteria. n, number

The hearing threshold and otologic evaluations were performed only in participants aged 40 years or older in the eighth and ninth Korea KNHANES because of time and cost constraints. Among the 8,526 participants aged 40 years or older (4,488 in 2020 and 4,038 in 2022), 6,918 individuals had available data on household income, household size, college graduation, current smoking, current drinking, strength training, hypertension, diabetes, history of otitis media, hearing thresholds at 0.5, 1, 2, and 4 kHz, tympanometry results, and tinnitus status. Among them, 6,094 participants with normal tympanometry findings and without a history of otitis media were selected. After excluding participants with acute tinnitus, a total of 5,965 participants were finally enrolled (3,140 in 2020 and 2,825 in 2022) (Fig. 1). After applying the sampling weights, these participants represented 21,253,241 individuals in 2020 and 21,078,382 individuals in 2022.

In South Korea, strict disease prevention measures, including social distancing, were implemented beginning on February 29, 2020. On November 1, 2021, the government initiated a phased return to normal daily life under a living-with-COVID-19 policy. Accordingly, participants were categorized based on the survey period: those surveyed in 2020 were assigned to the COVID-19 restriction period, and those surveyed in 2022 to the living-with-COVID-19 period.

The 8th and 9th Korea National Health and Nutrition Examination Survey (KNHANES), conducted in 2019–2021 and 2022–2023, respectively, were approved by the Institutional Review Board (IRB) of the Korea Disease Control and Prevention Agency (KDCA) (IRB No. 2018-01-03–2 C-A, 2022-11-16-R-A). Written informed consent was obtained from all participants in the survey. The present study, which used de-identified publicly available KNHANES data for secondary analysis, was exempted from additional IRB review by IRB of Hanyang University (IRB No. 2026-05-033).

### Otologic evaluation

Pure-tone audiometry was performed in a double-walled soundproof booth using an AD629 audiometer (Interacoustics, Assens, Denmark) to assess hearing thresholds. The mean hearing threshold was calculated by averaging thresholds at 0.5, 1, 2, and 4 kHz. Tympanic membrane status was evaluated through impedance audiometry using a Titan IMP440 screener (Interacoustics, Assens, Denmark), with a type A tympanogram regarded as normal. All audiologic assessments were conducted by four specially trained nurses certified by the Korean Society of Otorhinolaryngology–Head and Neck Surgery and supervised by otolaryngologists from the same organization.

Tinnitus was assessed based on the experience of tinnitus lasting for at least 5 min during the past year, and chronic tinnitus was defined as tinnitus persisting for 6 months or longer. Tinnitus severity was evaluated using a visual analog scale (VAS) ranging from 0 to 10, based on the question, “How bothersome is your tinnitus?”, with higher scores indicating greater tinnitus-related bother. Based on the previous study, patients with tinnitus and a VAS score of 0–4 were classified as the non-bothersome tinnitus group, whereas those with a VAS score of 5 or higher were classified as the bothersome tinnitus group [20].

### Depressive symptoms and stress evaluation

The Patient Health Questionnaire-9 (PHQ-9), comprising nine items corresponding to the DSM-based criteria for depressive disorders—including loss of interest, depressed mood, sleep disturbance, fatigue, appetite change, poor concentration, psychomotor change, feelings of worthlessness, and suicidal ideation—was used to evaluate depressive status [21]. Depressive symptoms were defined as a PHQ-9 score of 5 or higher, and depression was defined as a PHQ-9 score of 10 or higher; the latter cutoff was used for sensitivity analysis owing to the limited number of subjects in each tinnitus group [21].

Stress perception was evaluated through a single self-reported item asking participants to rate their perceived level of stress in daily life: “How much stress do you usually feel in your daily life?” Using the binary variable provided by the KNHANES, participants who endorsed elevated stress levels — specifically those responding “very much” or “a lot” — were categorized as the high-stress group.

### Statistical analysis

To ensure national representativeness of the general population in South Korea, sampling weights were applied and all analyses were performed using a complex sampling design. Differences in categorical variables were evaluated using complex sample cross-tabulation analyses, whereas continuous variables were compared using complex sample general linear models and are presented as means with standard errors (SE). For adjusted analyses, complex sample logistic regression was used for categorical outcomes, and complex linear regression was applied for continuous outcomes. Statistical significance was defined as a two-sided P value < 0.05. Bonferroni correction was used for post hoc pairwise comparisons. All statistical analyses were performed using IBM SPSS Statistics (IBM Corp., Armonk, NY, USA).

## Results

### Demographic factors,socioeconomic factors, medical history, otologic characteristics, and psychological health between COVID-19 restriction (2020) and living-with COVID-19 (2022) periods

Age, sex, household income, college graduation, current smoking, current drinking, strength training, hypertension, diabetes, hearing thresholds, chronic tinnitus, PHQ-9 scores, depressive symptoms, and high stress were compared between the COVID-19 restriction group and the living-with-COVID-19 group. Among these variables, only household size was greater in the COVID-19 restriction group (2.93 ± 0.04 vs. 2.66 ± 0.04, *p* < 0.001). For participants with chronic tinnitus, tinnitus severity did not differ between the groups (4.64 ± 0.21 vs. 4.12 ± 0.23 in living-with-COVID-19 group, *p* = 0.101). Otherwise, the other variables were comparable between the two periods (Table 1).

**Table 1:** Demographic characteristics, socioeconomic characteristics, underlying diseases, otologic profiles, and psychological status during the COVID-19 (2020) restriction period and the living-with-COVID-19 period (2022)

Variables	COVID-19 restriction (2020) (*n* = 3140/*n = 21,253,241)	Living-with-COVID-19 (2022) (*n* = 2,825/*n = 21,078,382)	*p*-value
Age (years)	56.80 ± 0.36 years	57.69 ± 0.39 years	0.091
Sex (female %)	51.78 ± 0.82%	51.65 ± 0.98%	0.917
Household income	3.37 ± 0.06	3.32 ± 0.05	0.463
1 st quintile	12.04 ± 1.00%	13.08 ± 0.93%	0.790
2nd quintile	16.30 ± 1.02%	17.54 ± 1.15%	
3rd quintile	20.61 ± 1.03%	20.16 ± 1.06%	
4th quintile	24.59 ± 1.20%	23.17 ± 1.22%	
5th quintile	26.45 ± 1.83%	26.06 ± 1.44%	
Household size	2.93 ± 0.04	2.66 ± 0.04	***< 0.001***
College graduation	37.45 ± 1.93%	41.05 ± 1.59%	0.153
Current smoking	19.23 ± 0.90%	19.54 ± 1.04%	0.817
Current drinking	68.65 ± 1.07%	70.64 ± 1.09%	0.193
Strength training (≥ 2 times/week)	21.70 ± 0.78%	22.60 ± 1.03%	0.485
Hypertension (%)	29.12 ± 1.10%	32.35 ± 1.37%	0.065
Diabetes (%)	12.55 ± 0.71%	13.84 ± 0.72%	0.201
Hearing thresholds (dB)	17.09 + ± 0.35 dB	17.79 ± 0.32 dB	0.143
Chronic tinnitus (%)	6.91 ± 0.49%	7.44 ± 0.59%	0.496
Tinnitus severity (VAS)^a^	4.64 ± 0.21	4.12 ± 0.23	0.100
PHQ-9	2.04 ± 0.08	2.11 ± 0.09	0.560
Depressive symptoms	14.93 ± 0.90%	15.69 ± 0.99%	0.568
High stress	24.63 ± 1.5	22.20 ± 1.06%	0.106

n, number; VAS, visual analogue scale; PHQ-9, Patient Health Questionnaire-9

*, Values were calculated after applying complex sample weights

^a^, Calculated only in chronic tinnitus patients

### Depressive symptoms and stress according to chronic tinnitus severity in between the COVID-19 Restriction (2020) and living-with-COVID-19 (2022) periods

In 2020, the numbers of participants with bothersome tinnitus, non-bothersome tinnitus, and no tinnitus were 144, 113, and 2,883, respectively, representing estimated population counts of 780,327, 689,132, and 19,783,781 individuals. In 2022, the corresponding figures were 121, 115, and 2,589 participants, representing 824,669, 742,544, and 19,511,169 individuals, respectively.

During the COVID-19 restriction period, PHQ-9 scores, depressive symptoms, and stress differed significantly among the bothersome tinnitus group, non-bothersome tinnitus group, and non-tinnitus group (*p* < 0.001 for PHQ-9 scores and depressive symptoms; *p* = 0.008 for high stress). In the post hoc analysis, the bothersome tinnitus group showed higher PHQ-9 scores (4.63 ± 0.56) than the non-tinnitus group (1.95 ± 0.08, *p* < 0.001) and the non-bothersome tinnitus group (1.68 ± 0.25, *p* < 0.001). In contrast, during the living-with-COVID-19 period, there were no significant differences in PHQ-9 scores (*p* = 0.098), depressive symptoms (*p* = 0.195), and stress levels (*p* = 0.437) among the bothersome tinnitus, non-bothersome tinnitus, and non-tinnitus group (Table 2). However, the bothersome tinnitus group showed higher PHQ-9 scores compared to the non-tinnitus group in the post-hoc test (*p* = 0.032).

**Table 2:** Comparison of Patient Health Questionnaire-9 (PHQ-9) scores, depressive symptoms (PHQ-9 ≥ 5), and high stress between the COVID-19 restriction period (2020) and the living-with-COVID-19 period (2022), stratified by tinnitus severity

Periods	COVID-19 restriction (2020) (*n* = 3,140/*n = 21,253,241)				Living-with-COVID-19 (2022) (*n* = 2,825/*n = 21,078,382)			
Groups	Non-tinnitus	Non-bothersome tinnitus	Bothersome-tinnitus	*p*-value	Non-tinnitus	Non-bothersome tinnitus	Bothersome-tinnitus	*p*-value
PHQ-9 (scores)	1.95 ± 0.08	1.68 ± 0.25	4.63 ± 0.56	***< 0.001***	2.08 ± 0.09	1.81 ± 0.37	3.14 ± 0.49	0.098
Depressive symptoms (%)	14.27 ± 0.91%	7.49 ± 2.46%	38.24 ± 4.62%	***< 0.001***	15.48 ± 1.02%	14.19 ± 3.88%	21.97 ± 3.82%	0.195
High stress (%)	24.24 ± 1.14%	20.21 ± 4.43%	38.30 ± 4.64%	***0.008***	22.34 ± 1.09%	17.00 ± 3.92%	23.69 ± 4.16%	0.437

n, number; PHQ-9, Patient Health Questionnaire-9

*, Values were calculated after applying complex sample weights

^a^, Calculated only in chronic tinnitus patients

**Fig. 2:**
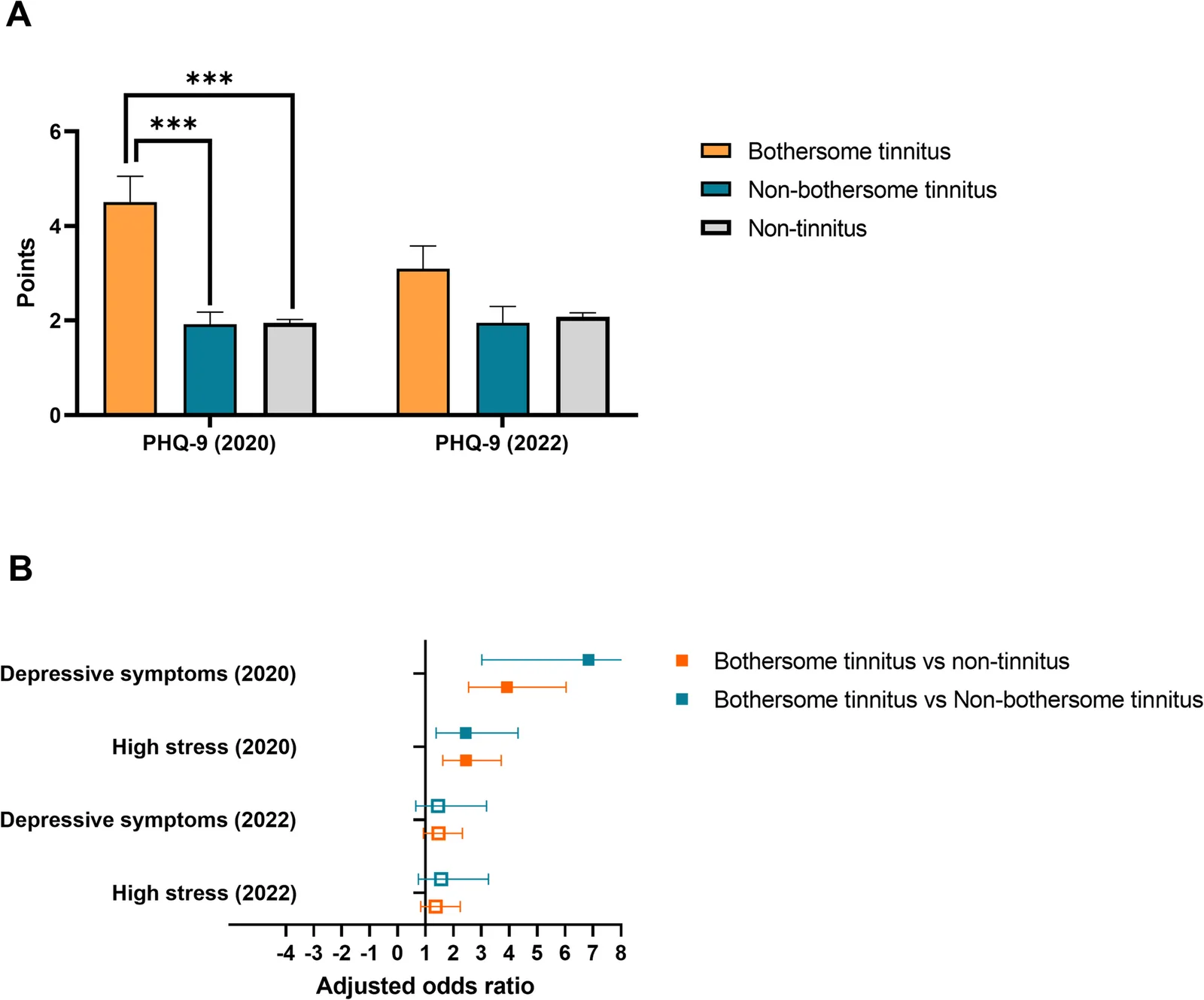
Multivariable analyses of Patient Health Questionnaire-9 (PHQ-9) scores, depressive symptoms, and high stress according to tinnitus severity during the COVID-19 restriction period (2020) and the living-with-COVID-19 period (2022). Analyses were adjusted for age, sex, household income, hypertension, diabetes, and hearing thresholds. (**A**) Multivariable analysis of PHQ-9 scores in patients stratified by tinnitus severity during the COVID-19 restriction period (2020) and the living-with-COVID-19 period (2022). (**B**) Multivariable analysis of the prevalence of depressive symptoms and high stress in patients stratified by tinnitus severity during the COVID-19 restriction period (2020) and the living-with-COVID-19 period (2022). Filled symbols indicate statistically significant differences. PHQ-9, Patient Health Questionnaire-9; ***, *p* < 0.001

In the multivariable analysis adjusting for age, sex, household income, household size, college graduation, current smoking, current drinking, strength training, hypertension, diabetes, and hearing thresholds, the bothersome tinnitus group also showed higher PHQ-9 scores (4.51 ± 0.55) and a higher prevalence of depressive symptoms and high stress than the non-tinnitus group (PHQ-9: 1.95 ± 0.08, *p* < 0.001; depressive symptoms: aOR = 3.915, 95% CI = 2.540–6.034, *p* < 0.001; high stress, aOR = 2.449, 95% CI = 1.616–3.712, *p* < 0.001) during the COVID-19 restriction period (Fig. 2A and B). Furthermore, the bothersome tinnitus group had higher PHQ-9 scores and prevalence of depressive symptoms and high stress than the non-bothersome tinnitus group (PHQ-9: 1.92 ± 0.26, *p* < 0.001; depressive symptoms: aOR = 6.834, 95% CI = 3.018–15.476, *p* < 0.001 high stress, aOR = 2.443, 95% CI = 1.384–4.313, *p* = 0.002) during the same period (Fig. 2A and B). In contrast, PHQ-9 scores, depressive symptoms, and high stress were not significantly different among the groups during the living-with-COVID-19 period (Fig. 2A and B).

###  Differences in depressive symptoms and stress according to chronic tinnitus severity between the COVID-19 restriction (2020) and living-with-COVID-19 (2022) periods

**Fig. 3:**
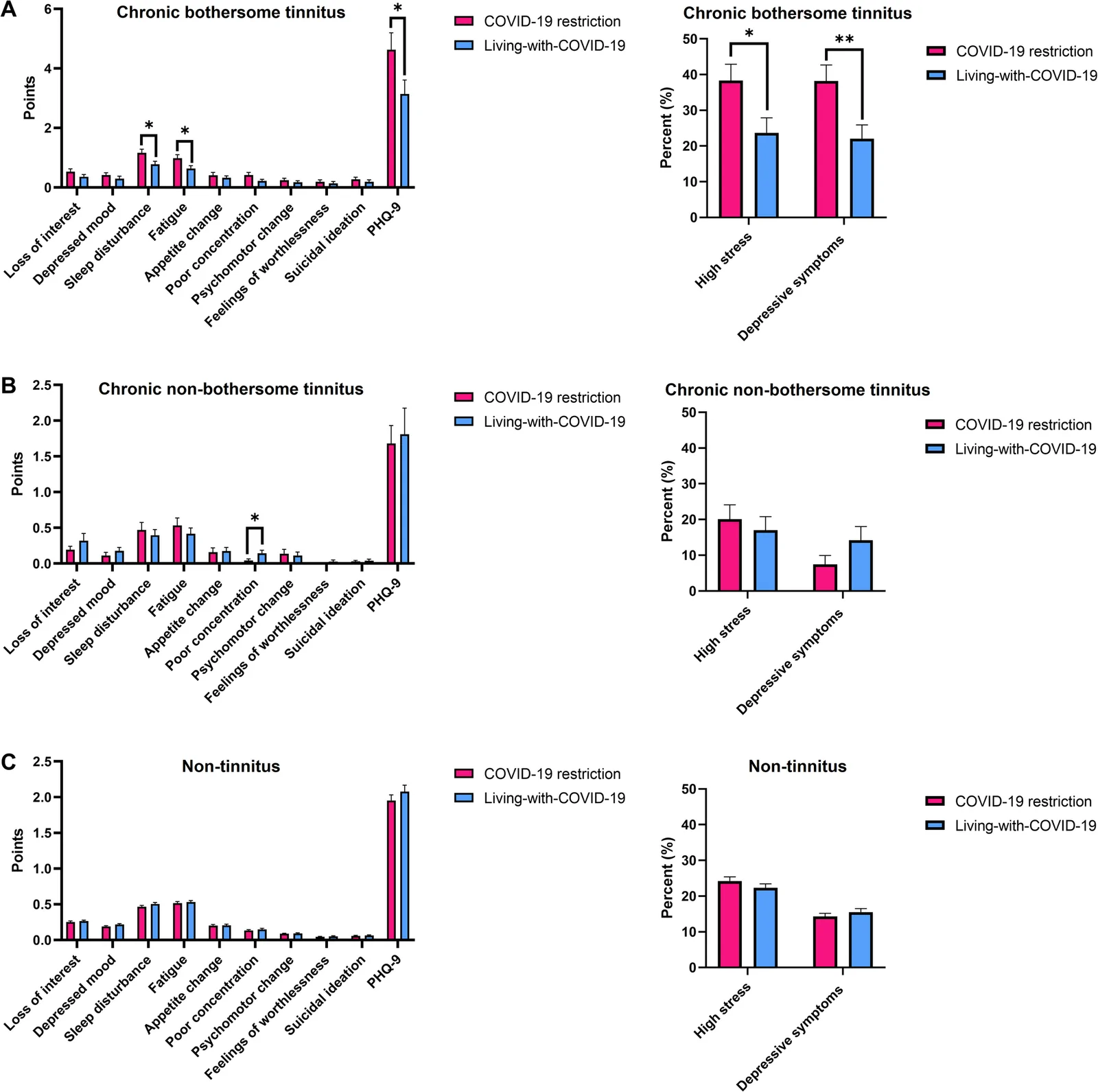
Comparison of depression (PHQ-9), depressive symptoms, and high stress between the COVID-19 restriction period and the living-with-COVID-19 period stratified by tinnitus distress. (**A**) Bothersome tinnitus, (**B**) non-bothersome tinnitus, and (**C**) non-tinnitus. PHQ-9, Patient Health Questionnaire-9; *, *p* < 0.05; **, *p *< 0.01

The differences in depressive symptoms and stress were analyzed within each tinnitus severity group between COVID-19 restriction and living-with-COVID-19 periods. In the bothersome tinnitus group, the PHQ-9 scores and prevalences of depressive symptoms and high stress were substantially higher during the COVID-19 restriction period (PHQ-9: 4.63 ± 0.57 vs. 3.14 ± 0.47, *p* = 0.045; depressive symptoms: 38.24 ± 4.45% vs. 21.97 ± 3.85%, *p* = 0.008; high stress: 38.30 ± 4.60% vs. 22.69 ± 4.21%, *p* = 0.023), while such patterns were not observed in the non-bothersome tinnitus or non-tinnitus groups (Fig. 3A and B, and 3C). Because both survey periods were nationally representative and thus broadly comparable across most demographic characteristics, with household size being the notable exception, multivariable analysis was performed adjusting for demographic factors, including age, sex, and household size. After this adjustment, differences in PHQ-9 scores and the prevalence of depressive symptoms and high stress between the COVID-19 restriction and living-with-COVID-19 periods remained significant (PHQ-9: 4.61 ± 0.56 vs. 3.16 ± 0.47, *p* = 0.048; depressive symptoms: aOR = 2.230, 95% CI = 1.228–4.050 *p* = 0.009; high stress: aOR = 2.052, 95% CI = 1.038–4.056, *p* = 0.039).

When further PHQ-9 subitems were compared between the two periods in the bothersome tinnitus group, sleep disturbance and fatigue were significantly more prevalent during the COVID-19 restriction period in both the univariable analysis (sleep disturbance: 1.16 ± 0.13 vs. 0.78 ± 0.10, *p* = 0.021; fatigue: 0.98 ± 0.12 vs. 0.64 ± 0.10, *p* = 0.028) (Fig. 3A) and the multivariable analysis adjusted for age, sex, and household size (sleep disturbance: 1.16 ± 0.12 vs. 0.78 ± 0.10, *p* = 0.021; fatigue: 0.98 ± 0.12 vs. 0.64 ± 0.10, *p* = 0.030).

### Sensitivity analysis for depression (PHQ-9 score ≥ 10)

To evaluate clinically relevant depression, further analysis for depression (PHQ-9 score ≥ 10) was conducted. The proportion of depression was significantly different among the bothersome tinnitus (13.66 ± 3.81%), non-bothersome tinnitus (2.24 ± 1.38%), and non-tinnitus groups (3.28 ± 0.42%) during the COVID-19 restriction period (*p* < 0.001), while this pattern was not observed during the living-with-COVID-19 period (*p* = 0.339). After adjusting for age, sex, household income, household size, college graduation, current smoking, current drinking, hypertension, diabetes, and hearing thresholds, depression was significantly more prevalent in the bothersome tinnitus group than in the non-tinnitus group (aOR = 3.992, 95% CI = 1.941–8.207, *p* < 0.001) or the non-bothersome tinnitus group (aOR = 6.033, 95% CI = 1.478–24.636, *p* < 0.001) during the COVID-19 restriction period.

An analysis was conducted to compare the prevalence of depression between the two study periods among participants with bothersome tinnitus. Although the prevalence was numerically higher during the COVID-19 restriction period (13.66 ± 3.89%) than during the living-with-COVID-19 period (6.60 ± 2.58%), the difference was not statistically significant (*p* = 0.125).

## Discussion

COVID-19 has exerted a wide-ranging influence on overall quality of life, as well as on physical and mental health outcomes at both the individual and societal levels. In the present study, we directed our analysis toward individuals with chronic tinnitus and found that those with bothersome tinnitus experienced a disproportionately higher burden of depressive symptoms and stress during the pandemic.

These findings indicate that individuals with bothersome tinnitus may be particularly associated with the adverse mental health consequences of large-scale disasters such as the COVID-19 pandemic and the social constraints imposed in response to them. Previous studies have reported aggravated depressive symptoms in tinnitus patients during the pandemic [22, 23]; however, results have been inconsistent, and a clear association with tinnitus severity has not been established [22–24]. The present study extends these findings by demonstrating that, when stratified by tinnitus severity, bothersome tinnitus was specifically associated with significantly greater depressive burden during the COVID-19 restriction period. This association may have been more apparent in the present study because South Korea implemented relatively extensive and prolonged social restriction measures during the pandemic, including strict social distancing and limitations on social activities, which may have been associated with psychological stress among vulnerable individuals [25, 26].

The elevated psychological burden observed in individuals with bothersome tinnitus during the COVID-19 restriction period may be explained by shared neurobiological mechanisms underlying both tinnitus-related distress and depressive symptoms [8–11, 14]. Dysregulation of the limbic system, particularly hyperactivation of the amygdala and hippocampal dysfunction, creates a self-reinforcing cycle in which tinnitus distress and depressive symptoms mutually exacerbate one another [14, 19]. Moreover, aberrant connectivity within the default mode network (DMN) promotes excessive rumination and attentional fixation on tinnitus [8–11], while overactivation of the salience network (SN) prevents habituation by persistently tagging the tinnitus percept as a threatening stimulus — patterns that are equally characteristic of depressive symptoms [8, 9, 12, 13]. At the neurochemical level, dysfunction of the serotonergic and noradrenergic systems, along with HPA axis dysregulation and reduced BDNF signaling, further converges to heighten vulnerability to affective dysregulation in this population [19, 27].

Given these overlapping neural substrates, it is possible that the chronic stress imposed by pandemic-related social restrictions was associated with greater psychological distress in individuals with bothersome tinnitus, potentially making them more vulnerable to depressive symptoms and perceived stress compared to those with non-bothersome tinnitus or without tinnitus altogether [1–3, 9, 19]. Furthermore, during the COVID-19 pandemic, appropriate treatment may not have been adequately provided because of various social restrictions, and reduced access to management strategies for tinnitus, depressive symptoms, and stress may have further aggravated these problems [28]. Therefore, careful consideration of the broader public health consequences of prolonged social restrictions is warranted, particularly regarding their psychiatric and psychological impact on vulnerable populations, including individuals with bothersome tinnitus. Furthermore, the development of diverse and accessible treatment protocols, including non-face-to-face approaches such as app-based software interventions incorporating sound therapy and cognitive behavioral therapy, may be necessary to ensure continuity of care during future public health crises [29–31]. Future prospective studies are needed to evaluate whether telemedicine- and app-based interventions can effectively reduce psychological distress in patients with bothersome tinnitus during public health emergencies.

Contrary to bothersome tinnitus, non-bothersome tinnitus did not show significant differences in psychological distress between the COVID-19 restriction period and the living-with-COVID-19 period. Non-bothersome tinnitus is often relatively habituated and carries lower emotional salience, making individuals less susceptible to additional psychological burden during the pandemic [19, 32]. In addition, tinnitus bothersomeness itself may partly reflect underlying individual differences in emotional vulnerability, stress coping mechanisms, and personality traits [19]. Therefore, beyond simply assessing the presence of tinnitus, evaluating tinnitus bothersomeness may provide additional insight into patients’ underlying psychological status and emotional vulnerability, and should be considered in clinical management and treatment planning.

Among the PHQ-9 subitems, sleep disturbance and fatigue showed the most prominent differences between the two periods. This finding may reflect the substantial lifestyle disruption, social isolation, and stress-related hyperarousal associated with the COVID-19 restriction period, which are closely linked to sleep quality and daytime fatigue [18, 19, 33–36]. Furthermore, the relationship between sleep disturbance and tinnitus is likely bidirectional; disrupted sleep may have exacerbated tinnitus perception, while worsening tinnitus may have in turn further impaired sleep quality [35, 37]. In addition, depressive symptom itself has a bidirectional relationship with sleep disturbance and fatigue, and these symptoms are also partially reflected in the assessment of depressive symptoms [38, 39]. Therefore, the complex interplay among sleep disturbance, tinnitus, and depressive symptoms requires further investigation. Nevertheless, sleep disturbance represents a substantial clinical concern among patients with tinnitus, particularly during the COVID-19 pandemic, and previous studies have demonstrated that sleep quality can influence both tinnitus severity and depressive symptoms [35, 39–41]. Therefore, despite the need for further clarification of their interrelationships, sleep-focused interventions — such as sleep hygiene education, and cognitive behavioral therapy for insomnia (CBT-I) — should be considered as an important component of comprehensive care for patients with tinnitus and psychological distress [40].

Although the proportion of depression (PHQ-9 scores ≥ 10) appeared to be higher among participants with bothersome tinnitus during the COVID-19 restriction period than during the living-with-COVID-19 period, the between-period difference was not statistically significant. This finding was likely attributable to the limited sample size of participants with bothersome tinnitus and depression (*n* = 18 in 2020 and *n* = 8 in 2022), which reduced the statistical power to detect a significant difference. Further studies with a larger sample of participants with depression are warranted.

This study has several limitations. First, because the present study was conducted using a public database, the use of additional validated assessment tools for tinnitus and depressive symptoms, such as the Tinnitus Handicap Inventory (THI), Tinnitus Functional Index (TFI), Beck Depression Inventory (BDI), or Hamilton Depression Rating Scale (HAM-D), was limited [5–7, 42, 43]. Unlike the THI, which consists of domain-specific subscales assessing functional, emotional, and catastrophic aspects of tinnitus, the VAS captures only the overall subjective distress associated with tinnitus through a single global score. Consequently, this study was limited in its ability to identify the specific domains contributing to tinnitus-related distress, and a comprehensive evaluation of tinnitus-related distress was not feasible. Second, although this study used a nationally representative dataset that enhances the generalizability of the findings, causal relationships between psychological distress and tinnitus could not be determined due to the repeated cross-sectional design. Third, treatment modalities for tinnitus or depressive symptoms could not be considered because of limited available information. Fourth, as the study was based on survey data, it is inherently subject to recall bias. Fifth, differences observed between the two survey periods may have been influenced by unmeasured factors, including COVID-19 vaccination, changes in the social environment, healthcare access, and other secular trends. Therefore, these contextual factors may have contributed to the observed findings. Further prospective studies with large sample sizes are warranted to better clarify these associations.

## Conclusion

During the COVID-19 pandemic era, bothersome chronic tinnitus showed a significant association with an increased prevalence of depressive symptoms and high stress. Given the limited access to medical care during the pandemic, careful and strategic management of tinnitus and associated emotional distress using non-face-to-face approaches, such as telemedicine or application-based software technologies, should be considered. Furthermore, in patients with tinnitus, assessment of tinnitus severity may help identify underlying emotional vulnerability and should be taken into consideration in clinical management and treatment planning.

## Data Availability

The data used in this study.

## References

[1] Serafini G, Parmigiani B, Amerio A, Aguglia A, Sher L, Amore M (2020) The psychological impact of COVID-19 on the mental health in the general population. QJM 113(8):531–537. 10.1093/qjmed/hcaa201 PMC7337855 32569360

[2] Saladino V, Algeri D, Auriemma V (2020) The Psychological and Social Impact of Covid-19: New Perspectives of Well-Being. Front Psychol 11–2020. 10.3389/fpsyg.2020.577684 PMC7561673 33132986

[3] Wang SM, Kim SH, Choi WS, Lim HK, Woo YS, Pae CU, Bahk WM (2023) The Impact of COVID-19 on Psychiatric Health in the Korean Population. Clin Psychopharmacol Neurosci 21(3):410–418. 10.9758/cpn.23.1083 PMC10335912 37424410

[4] Kim MS, Kim K, Lee K, Nehs CJ, Pizzol D, Kang J, Yon DK (2026) Trends in physical activity among South Korean adults before, during, and after the COVID-19 pandemic, 2009–2024: A nationwide representative study. J Sci Med Sport. 10.1016/j.jsams.2026.01.001 41592974

[5] Han BI, Lee HW, Kim TY, Lim JS, Shin KS (2009) Tinnitus: characteristics, causes, mechanisms, and treatments. J Clin Neurol 5(1):11–19. 10.3988/jcn.2009.5.1.11 PMC2686891 19513328

[6] Langguth B, Kreuzer PM, Kleinjung T, De Ridder D (2013) Tinnitus: causes and clinical management. Lancet Neurol 12(9):920–930. 10.1016/s1474-4422(13)70160-1 23948178

[7] Lee HY, Jung DJ (2023) Recent Updates on Tinnitus Management. J Audiol Otol 27(4):181–192. 10.7874/jao.2023.00416 PMC10603282 37872753

[8] De Ridder D, Vanneste S, Song JJ, Adhia D (2022) Tinnitus and the triple network model: a perspective. Clin Exp Otorhinolaryngol 15(3):205–212. 10.21053/ceo.2022.00815 PMC9441510 35835548

[9] Lee SJ, Park J, Lee SY, Koo JW, Vanneste S, De Ridder D, Lim S, Song JJ (2022) Triple network activation causes tinnitus in patients with sudden sensorineural hearing loss: a model-based volume-entropy analysis. Front Neurosci 16:1028776. 10.3389/fnins.2022.1028776 PMC9714300 36466160

[10] Cheng W, Rolls ET, Qiu J, Xie X, Wei D, Huang C-C, Yang AC, Tsai S-J, Li Q, Meng J, Lin C-P, Xie P, Feng J (2018) Increased functional connectivity of the posterior cingulate cortex with the lateral orbitofrontal cortex in depression. Translational Psychiatry 8(1):90. 10.1038/s41398-018-0139-1 PMC5915597 29691380

[11] Ho TC, Connolly CG, Henje Blom E, LeWinn KZ, Strigo IA, Paulus MP, Frank G, Max JE, Wu J, Chan M, Tapert SF, Simmons AN, Yang TT (2015) Emotion-Dependent Functional Connectivity of the Default Mode Network in Adolescent Depression. Biol Psychiatry 78(9):635–646. 10.1016/j.biopsych.2014.09.002 PMC4362932 25483399

[12] Fox MD, Raichle ME (2007) Spontaneous fluctuations in brain activity observed with functional magnetic resonance imaging. Nat Rev Neurosci 8(9):700–711. 10.1038/nrn2201 17704812

[13] Schumer MC, Bertocci MA, Aslam HA, Graur S, Bebko G, Stiffler RS, Skeba AS, Brady TJ, Benjamin OE, Wang Y, Chase HW, Phillips ML (2024) Patterns of Neural Network Functional Connectivity Associated With Mania/Hypomania and Depression Risk in 3 Independent Young Adult Samples. JAMA Psychiatry 81(2):167–177. 10.1001/jamapsychiatry.2023.4150 PMC10620679 37910117

[14] Besteher B, Gaser C, Ivanšić D, Guntinas-Lichius O, Dobel C, Nenadić I (2019) Chronic tinnitus and the limbic system: Reappraising brain structural effects of distress and affective symptoms. Neuroimage Clin 24:101976. 10.1016/j.nicl.2019.101976 PMC6734051 31494400

[15] Salazar JW, Meisel K, Smith ER, Quiggle A, McCoy DB, Amans MR (2019) Depression in Patients with Tinnitus: A Systematic Review. Otolaryngol Head Neck Surg 161(1):28–35. 10.1177/0194599819835178 PMC7721477 30909841

[16] Betz LT, Mühlberger A, Langguth B, Schecklmann M (2017) Stress reactivity in chronic tinnitus. Sci Rep 7(1):41521. 10.1038/srep41521 PMC5278380 28134346

[17] Mazurek B, Boecking B, Brueggemann P (2019) Association Between Stress and Tinnitus-New Aspects. Otol Neurotol 40(4):e467–e473. 10.1097/mao.0000000000002180 30870382

[18] Mazurek B, Haupt H, Olze H, Szczepek AJ (2012) Stress and tinnitus-from bedside to bench and back. Front Syst Neurosci 6:47. 10.3389/fnsys.2012.00047 PMC3371598 22701404

[19] Patil JD, Alrashid MA, Eltabbakh A, Fredericks S (2023) The association between stress, emotional states, and tinnitus: a mini-review. Front Aging Neurosci 15:1131979. 10.3389/fnagi.2023.1131979 PMC10188965 37207076

[20] Han SY, Seo HW, Lee SH, Chung JH (2026) Tinnitus severity and chronicity in relation to hearing loss: a population-based study. Eur Arch Otorhinolaryngol 283(4):2309–2317. 10.1007/s00405-025-09978-6 41566031

[21] Kroenke K, Spitzer RL, Williams JB (2001) The PHQ-9: validity of a brief depression severity measure. J Gen Intern Med 16(9):606–613. 10.1046/j.1525-1497.2001.016009606.x PMC1495268 11556941

[22] Schlee W, Hølleland S, Bulla J, Simoes J, Neff P, Schoisswohl S, Woelflick S, Schecklmann M, Schiller A, Staudinger S, Probst T, Langguth B (2020) The Effect of Environmental Stressors on Tinnitus: A Prospective Longitudinal Study on the Impact of the COVID-19 Pandemic. J Clin Med 9(9). 10.3390/jcm9092756 PMC7565885 32858835

[23] Fioretti A, Natalini E, Triggianese G, Eibenstein R, Angelone AM, Lauriello M, Eibenstein A (2022) Impact of the COVID-19 Lockdown on Patients with Chronic Tinnitus-Preliminary Results. Audiol Res 12(3):327–336. 10.3390/audiolres12030034 PMC9220770 35735367

[24] Tai Y, Jain N, Kim G, Husain FT (2024) Tinnitus and COVID-19: effect of infection, vaccination, and the pandemic. Front Public Health 12:1508607. 10.3389/fpubh.2024.1508607 PMC11629081 39659719

[25] Heo G, Apio C, Han K, Goo T, Chung HW, Kim T, Kim H, Ko Y, Lee D, Lim J, Park T (2021) Statistical Estimation of Effects of Implemented Government Policies on COVID-19 Situation in South Korea. Int J Environ Res Public Health 18(4). 10.3390/ijerph18042144 PMC7926904 33671746

[26] Apio C, Han K, Lee D, Lee B, Park T (2024) Development of New Stringency Indices for Nonpharmacological Social Distancing Policies Implemented in Korea During the COVID-19 Pandemic: Random Forest Approach. JMIR Public Health Surveill 10:e47099. 10.2196/47099 PMC10775907 38190233

[27] Miranda V, Castiglioni S, Maier JA (2025) Neuroplasticity and tinnitus: the role of Brain-Derived Neurotrophic Factor in pathogenesis and treatment. Front Neurosci 19. 10.3389/fnins.2025.1620894 PMC12279856 40697272

[28] Hwang J, Kim W-H, Heo J (2023) An association between individual’s risk perceptions and delayed or foregone healthcare services during the COVID-19 pandemic in Korea. BMC Health Serv Res 23(1):850. 10.1186/s12913-023-09807-8 PMC10422804 37568127

[29] Han SY, Kim SM, Bae J, Lee J, Oh S, Kim YH (2026) Initial compliance as a predictor of therapeutic outcomes in chronic tinnitus. Med (Baltim) 105(2):e47147. 10.1097/md.0000000000047147 PMC12795005 41517757

[30] Engelke M, Simões J, Vogel C, Schoisswohl S, Schecklmann M, Wölflick S, Pryss R, Probst T, Langguth B, Schlee W (2023) Pilot study of a smartphone-based tinnitus therapy using structured counseling and sound therapy: A multiple-baseline design with ecological momentary assessment. PLOS Digit Health 2(1):e0000183. 10.1371/journal.pdig.0000183 PMC9931272 36812641

[31] Bernstein EE, Weingarden H, Wolfe EC, Hall MD, Snorrason I, Wilhelm S (2022) Human Support in App-Based Cognitive Behavioral Therapies for Emotional Disorders: Scoping Review. J Med Internet Res 24(4):e33307. 10.2196/33307 PMC9034419 35394434

[32] Cima RFF (2018) Bothersome tinnitus: Cognitive behavioral perspectives. Hno 66(5):369–374. 10.1007/s00106-018-0502-9 PMC5928170 29713758

[33] Azizi-Zeinalhajlou A, Mirghafourvand M, Nadrian H, Samei Sis S, Matlabi H (2022) The contribution of social isolation and loneliness to sleep disturbances among older adults: a systematic review. Sleep Biol Rhythms 20(2):153–163. 10.1007/s41105-022-00380-x PMC10900038 38469248

[34] Qi X, Pei Y, Malone SK, Wu B (2023) Social Isolation, Sleep Disturbance, and Cognitive Functioning (HRS): A Longitudinal Mediation Study. J Gerontol Biol Sci Med Sci 78(10):1826–1833. 10.1093/gerona/glad004 PMC10562894 36617184

[35] Han SY, Seo HW, Lee SH, Chung JH (2025) Relationship Between Chronicity and Severity of Tinnitus and Sleep-Related Issues. Otol Neurotol 46(8):991–997. 10.1097/mao.0000000000004552 40423717

[36] Kalmbach DA, Anderson JR, Drake CL (2018) The impact of stress on sleep: Pathogenic sleep reactivity as a vulnerability to insomnia and circadian disorders. J Sleep Res 27(6):e12710. 10.1111/jsr.12710 PMC7045300 29797753

[37] Awad M, Abdalla I, Jara SM, Huang TC, Adams ME, Choi JS (2024) Association of sleep characteristics with tinnitus and hearing loss. OTO Open 8(1):e117. 10.1002/oto2.117 PMC10900921 38420352

[38] Nutt D, Wilson S, Paterson L (2008) Sleep disorders as core symptoms of depression. Dialogues Clin Neurosci 10(3):329–336. 10.31887/DCNS.2008.10.3/dnutt PMC3181883 18979946

[39] Fang H, Tu S, Sheng J, Shao A (2019) Depression in sleep disturbance: A review on a bidirectional relationship, mechanisms and treatment. J Cell Mol Med 23(4):2324–2332. 10.1111/jcmm.14170 PMC6433686 30734486

[40] Scott AJ, Webb TL, Martyn-St James M, Rowse G, Weich S (2021) Improving sleep quality leads to better mental health: A meta-analysis of randomised controlled trials. Sleep Med Rev 60:101556. 10.1016/j.smrv.2021.101556 PMC8651630 34607184

[41] Marks E, Hallsworth C, Vogt F, Klein H, McKenna L (2023) Cognitive behavioural therapy for insomnia (CBTi) as a treatment for tinnitus-related insomnia: a randomised controlled trial. Cogn Behav Ther 52(2):91–109. 10.1080/16506073.2022.2084155 35762946

[42] Newman CW, Jacobson GP, Spitzer JB (1996) Development of the Tinnitus Handicap Inventory. Arch Otolaryngol Head Neck Surg 122(2):143–148. 10.1001/archotol.1996.01890140029007 8630207

[43] Seemüller F, Schennach R, Musil R, Obermeier M, Adli M, Bauer M, Brieger P, Laux G, Gaebel W, Falkai P, Riedel M, Möller HJ (2023) A factor analytic comparison of three commonly used depression scales (HAMD, MADRS, BDI) in a large sample of depressed inpatients. BMC Psychiatry 23(1):548. 10.1186/s12888-023-05038-7 PMC10386606 37507656

